# Is Mental Privacy a Component of Personal Identity?

**DOI:** 10.3389/fnhum.2021.773441

**Published:** 2021-10-14

**Authors:** Abel Wajnerman Paz

**Affiliations:** Department of Philosophy, Universidad Alberto Hurtado, Santiago, Chile

**Keywords:** neurorights, privacy, mental privacy, identity, relational identity, narrative identity

## Introduction

Motivated by the aim to minimize the significant suffering and economic impact caused worldwide by brain diseases and disorders such as dementia, chronic pain, depression, addiction, and autism, several national, and regional research projects emerged in the recent years to improve our understanding of brain function and dysfunction. These projects contributed to the development of neurotechnologies and techniques that have an unprecedented ability (both in terms of scope and reliability) to “read” mental states, in the sense of decoding information about mental states or processes by analyzing data about neural activity patterns, and “transcribe” mental states by modulating neural computation. These technologies and the knowledge obtained through them are critical to enabling novel therapies for brain disorders and therefore are ethically motivated by the bioethical principle of beneficence. However, it has been argued that they also entail new neuroethical challenges that have to be addressed at a regulatory level.

Recently, Yuste et al. (2021) suggested that existing international human rights need to be further expanded and/or specified because they address certain ethically relevant dimensions of human life in very generic terms, often subject to interpretation, and regulating the ramifications of neurotechnology requires greater specificity. Following this idea, countries such as Chile, Brazil and Spain are stablishing specific rights for the regulation of neurotechnology, also known as “neurorights.” Some of these proposals are inspired by the framework developed by the *Morningside Group* (an interdisciplinary group led by the neuroscientist Rafael Yuste), which introduces five key neurorights: the right to personal identity, the right to free will, the right to mental privacy, the right to equal access to cognitive enhancement technologies, and the right to protection against algorithmic bias.

One of the most discussed neurorights is probably *mental privacy*. This is the idea that we should have control over access to our neural data and to the information about our mental processes and states that can be obtained by analyzing it. The “mind-reading” neurotechnologies that could put mental privacy at risk consist of a wide variety of applications, including not only the interpretation of neural activity patterns in isolation (e.g., determining what I am thinking about without using any external cue), but also the use of neural responses to consciously perceived stimuli (e.g., P300 signals) for identifying experiences of recognition (Rissman et al., 2010), and the use of subliminal stimuli for detecting sexual preferences (Wernicke et al., 2017) and empathic responses (Chiesa et al., 2017).

A key issue is whether mental privacy is different from other forms of privacy. Specifically, we need to determine whether neural data and mental information needs more stringent protection than other kinds of personal information. I will articulate and support a proposal suggesting that mental privacy requires a special treatment because of its relation to relevant aspects of *personal identity*.

## Privacy as a Psychological Capacity

A recent proposal posits that mental privacy requires special legal protection because it is intimately related to who we are. More specifically, the Morningside Group's framework, crystalized in the recent Chilean neuroprotection bill, affirms that neural data should be treated as organic tissue and thus protected by the laws for organ transplantation and donation. This approach consists of two main aspects. Firstly, people not only have a right to not be compelled to give up brain data but, crucially, brain data collection requires explicit “opt-in” authorization. Secondly, brain data cannot be commercially transferred and used but only donated for altruistic purposes. That is, the commercialization brain data is prohibited regardless of consent status (Yuste et al., 2017; Goering et al., 2021).

The appeal of this proposal depends on being able to explain exactly how mental privacy is related to who we are. A possible conceptualization of mental privacy as a *cognitive* dimension of privacy seems to bring it closer to our identity, thus perhaps grounding the Morningside group's view. Given that all forms of privacy depend ultimately on mentally processing personal information, this ability can be regarded as the cognitive source of privacy (Ienca and Andorno, 2017; Goering et al., 2021; Wajnerman Paz, 2021). Privacy is partly defined by the *control* that persons have over the flow of information about them (i.e., being able to determine “when, how, and to what extent information about them is communicated to others,” Westin, 1968). Most often, we say that information is under our control, at least in part, in a *cognitive* sense. For any piece of information, we can consciously grasp it, reason about its personal and social meaning and its potential applications, and finally decide whether, when, how, to whom and to which extent we want to share it. Privacy depends on this cognitive process of rationally filtering and selectively sharing information about us. Neurotechnological mind-reading may be specially threatening for privacy precisely because it by-passes this fundamental filtering process. Any given piece of information we have mentally considered and decided not to share will be anyway available to someone who has direct access to our mind. Technological mind-reading can potentially make the cognitive mechanisms that define privacy itself meaningless. Thus, by characterizing mental privacy as a psychological (and fundamental) dimension of privacy we can identify the risks it involves. However, a critical question is whether these risks affect some fundamental human dimension that is not threatened by other kinds of privacy violations.

This kind of cognitive approach to privacy has been developed in the environmental psychology literature. Interestingly, a prominent view entails that this capacity is part of what constitutes our *personal identity*. One of the most influential approaches to privacy as a psychological capacity is perhaps Irwin Altman's idea that privacy is a boundary regulation process (e.g., Altman, 1975). Altman characterizes privacy as the regulation of social interaction, aimed at achieving an ideal level of interpersonal contact by avoiding two kinds of imbalances: being unable to seclude ourselves (e.g., crowding) and overachieving seclusion (i.e., isolation). Crucially, this equilibrium depends on control over social inputs (e.g., actively accepting or rejecting other's opinions) and social outputs (e.g., avoid or allow someone listen to one's opinions) (Altman, 1976; see Margulis, 2003 for a review of Altman's approach).

Altman argued that the main function of this process is the construction of our identity, our understanding of ourselves. It includes “knowing where one begins and ends vis-a-vis others, what aspects of the physical and social environment are parts of the self and which aspects are parts of others” (Altman, 1976, p. 25). The notion of privacy implies a flexible barrier or boundary between the self and non-self, “such as the cell membrane becomes more or less permeable, in order to achieve a viable level of functioning” (Ibid, p. 13). However, it is not entirely clear whether this notion of privacy can be relevant for a conception of identity that may be useful in contemporary neuroethics. I will suggest that a recent and prominent approach to neurotechnological influences on identity seems to line up with key aspects of Altman's proposal.

## Discussion: Mental Privacy and Relational Identity

There has been a debate in the recent neuroethics literature regarding how we should conceptualize identity in the situations in which it seems to be threatened by “mind-writing” neurotechnologies, such as deep brain stimulation (DBS) and other devices that can potentially modulate neural computation (e.g., see Klein et al., 2016; Goering et al., 2017; Gilbert et al., 2018). Mackenzie and Walker (2015) argued that these threats should be characterized as threats to a narrative and relational conception of identity. I will claim that such notion is closely related to mental privacy as a psychological capacity.

Most identity-related concerns of DBS patients are plausibly not about the modification of our metaphysical essence or our individuation conditions, which are characterized by psychological and biological continuity theories of identity. The main worry seems to be better conceptualized in terms of practical identity, which articulates one's self-conception: one's defining beliefs and values, motives, emotions, etc. Furthermore, Mackenzie and Walker's view is that this self-conception is not discovered but rather *created*, there is no pre-existing real inner self awaiting to be found. We build our personal identities by developing *self-narratives*. These are cognitive structures through which we interpret our personal histories and psychological traits and shape our intentions and plans. Finally, Mackenzie and Walker (2015), characterize this self-creation process in a relational way. Following Baylis, they affirm that we are “dynamic complex co-creations informed by the perspectives and creative intentions of others” (Baylis, 2012, p. 118). Constructing a narrative identity is therefore an ongoing negotiation between our self-ascriptions of identity and the interpretation (or mis-interpretation) and recognition (or rejection) of these ascriptions by others. Baylis claims that a self-narrative is identity constituting only when it achieves a temporary stability between our perspective and that of relevant others, which depends on ongoing interpersonal communication.

If we understand identity as the result of this kind of interpersonal communicative process, then it is clear how psychological privacy in Altman's sense can be considered a constitutive part of it. The inter-subjective equilibrium in the construction of an agent's self-narrative requires her to regulate a communicative output constituted by her projected self-narrative and an input constituted by her perceived narrative (the recognition and/or interpretation of her self-attributions by others) in a way that reflects Altman's boundary regulation. We must first open our inter-personal “output gate” to make our self-narrative accessible to others, and also open our inter-personal “input gate,” being receptive to its interpretation and assessment by others, thus building a collectively agreed self-narrative. Crucially, when the self-narrative projected by an agent is disputed by others, she can achieve equilibrium through a variety of processes described by Baylis (2012), which also involve specific forms of inter-personal boundary regulation. She can seclude herself from dissonant interpretations by changing her community of belonging in favor of a community likely to be more accepting of her projected self-narrative, thus reshaping her inter-personal boundaries. Alternatively, the agent can change her output, trying to project her self-narrative more efficiently by sharing more information and being more open to others. Finally, she could be simply become more permeable to other's interpretations, accepting those that are inconsistent with her perspective and consequently revising her projected self-narrative.

Thus, the regulation of interpersonal interaction that is constitutive of an agent's mental privacy seems to be part of the communicative process that constitutes her identity. This means that violations of a person's mental privacy, disrupting the cognitive control she has over what information about herself she shares or receives may actually affect the very process underlying the formation of her identity.

## Conclusion

Mental privacy has emerged in the last years as a critical concern regarding emerging neurotechnologies. The threat is perhaps enhanced by the fact that these technologies increasingly exceed the clinical context. Many novel non-invasive and potentially ubiquitous consumer neurotechnologies have various educational, entertainment-related, work-related, and military applications, which are not fully explored or regulated by either national laws or international treaties.

Some of the pioneering regulatory frameworks and their applications by policy-makers support a strong reading of mental privacy, as a distinctive privacy dimension closely related to our identity. In this article, I suggested that this conceptual connection may be a key to deepen our understanding of mental privacy and articulate it with broader discussions in neuroethics. If we understand mental privacy as the psychological basis of privacy, we can see how it underlies the construction of our relational identity. Thus, relational identity may become a concern which should be addressed not only in relation to mind-writing neurotechnologies but also to neurotechnological mind-reading.

## Author Contributions

The author confirms being the sole contributor of this work and has approved it for publication.

## Funding

This work was supported by the Facultad de Filosofía y Humanidades, Universidad Alberto Hurtado and the Fondo Nacional de Desarrollo Cientco y Tecnolo (Proyecto Fondecyt Regular #1200197 and #1210091).

## Conflict of Interest

The author declares that the research was conducted in the absence of any commercial or financial relationships that could be construed as a potential conflict of interest.

## Publisher's Note

All claims expressed in this article are solely those of the authors and do not necessarily represent those of their affiliated organizations, or those of the publisher, the editors and the reviewers. Any product that may be evaluated in this article, or claim that may be made by its manufacturer, is not guaranteed or endorsed by the publisher.
